# Genotyping of *BRCA1, BRCA2, p53, CDKN2A*, *MLH1* and *MSH2* genes in a male patient with secondary breast cancer

**DOI:** 10.2478/v10019-011-0031-6

**Published:** 2011-09-22

**Authors:** Ana Lina Vodusek, Srdjan Novakovic, Vida Stegel, Berta Jereb

**Affiliations:** 1 Department of Radiation Oncology, Institute of Oncology, Ljubljana, Slovenia; 2 Department of Molecular Diagnostics, Institute of Oncology, Ljubljana, Slovenia

**Keywords:** gene screening, breast cancer, male, secondary neoplasm

## Abstract

**Background:**

Some tumour suppressor genes (*BRCA2*) and mismatch repair genes (*MSH2, MLH1*) are correlated with an increased risk for male breast cancer.

**Case report:**

Our patient developed secondary breast cancer after the treatment for Hodgkin’s disease in childhood. DNA was isolated from the patients’ blood and screened for mutations, polymorphisms and variants in *BRCA1, BRCA2, p53, CDKN2A*, *MLH1* and *MSH2* genes. We found no mutations but common polymorphisms, and three variants in mismatch repair genes.

**Conclusions:**

Nucleotide variants c.2006-6T>C and p.G322D in *MSH2* might be correlated with male breast cancer.

## Introduction

Secondary neoplasms (SN) are the most serious late effects of the treatment of childhood cancers. Their incidence is increasing with time of observation to 25% at 25 years from diagnosis of the primary tumor.^1^,^2^ Patients treated with radiotherapy for Hodgkin’s disease (HD) are at highest risk for SN.^3^

Male breast cancer (MBC) is rare, accounting for less than 1% of all breast cancers.^4^,^5^ Risk factors for primary MBC include testicular disease, benign breast conditions, age, family history, the Klinefelter syndrome, gynecomasty and non-therapeutic radiation exposure.^5^,^6^ The highest risk for primary MBC is among the carriers of mutations in the *BRCA2* gene. Besides mutations in the *BRCA2* gene, additional germ line mutations in MBC have been reported also in the androgen receptor gene and *PTEN*.^7^ There is likely to be a number of genes more commonly mutated correlated with a modest increase in primary MBC, such as mismatch repair (MMR) genes - *MSH2, MLH1, PMS1, PMS2*.^7^,^8^

In literature we found two case reports on secondary MBC, one after the treatment for HD and one after the treatment for acute lymphoblastic leukaemia including total body irradiation and bone marrow transplantation.^3^,^9^ There are no data on genetic factors predisposing the development of secondary MBC after the treatment for HD.

The aim of this case report was to elucidate the genetic conditions in a patient with secondary MBC, screening the genes already known to be correlated with primary MBC - *BRCA1, BRCA2, p53, CDKN2A*, *MLH1* and *MSH2.*

## Case report

### Patient

A 37 year old man developed breast cancer 24 years after the treatment for HD. In 1983, when 13 years old, he was treated with chemotherapy (6 cycles of MOPP/ABVD) and irradiation of the neck and upper thorax, retroperitoneal and inguinal nodes (25 Gy) for stage IIIBS HD. Afterwards he was followed, at the Department of Paediatrics and after 1995 at the Late Effect Clinic at the Institute of Oncology in Ljubljana.

Eleven years after the treatment we observed primary hypogonadism with low levels of testosterone, bilateral gynecomasty and azoospermia and at the age of 37 a palpable tumour 6 cm in diameter in the central part of the left breast, infiltrating the skin, with an inverted nipple. Ultrasounds of both axillae, of the abdomen, chest X-ray films and Technetium bone scan were normal.

Histology showed an invasive ductal carcinoma, grade III, with negative progesterone and estrogen receptors and positive Her2 status. His family history was negative. He was treated with neoadjuvant chemotherapy, left mastectomy with axillary node dissection, adjuvant treatment with trastuzumab and postoperative irradiation of the left mammary region.

### Methods of genetic investigations

Patient’s DNA was isolated from peripheral blood using the DNA blood isolation kit Quiagen (Hilden, Germany). It was screened for variants in tumour suppressor genes (*BRCA1, BRCA2, p53, CDKN2A*) and MMR genes (*MLH1* and *MSH2*).

Four methods were used: DGGE (denaturizing gradient gel electrophoresis) or HRM (high resolution melting), direct sequencing and MLPA (multiplex ligation-dependent probe amplification). The screening of *BRCA1/2* genes was performed for all exons by the DGGE while *MLH1* and *MSH2* genes were screened using the HRM. Positive fragments were subsequently sequenced to determine the nucleotide change. Genes *BRCA1, BRCA2, MLH1* and *MSH2* were also screened for large deletions or insertions using the MLPA method. All coding regions of *p53* and *CDKN2A* were sequenced.

**HRM** was used for discrimination between two DNA molecules with different sequences for the detection of SNPs (single nucleotide polymorphism) and small deletions and insertions. PCR (polymerase chain reaction) was performed on LC480 instrument using the LC 480 High-resolution Melting Master Kit (Roche, Manheim, Germany) according to manufacturer’s instructions.

**DGGE** was used for detection of SNPs and small deletions or insertions. The PCR amplification of DNA samples was performed using a set of GC-clamped primers (Ingeny International BV, Goes, Netherlands) according to the cycling conditions provided by the primer-manufacturer. Different denaturing and running conditions were used for specific primer sets. When electrophoresis was complete, gels were stained with EtBr and documented using the GelDoc system.

**Direct sequencing** was performed using the ABI PRISM Big Dye Terminator Cycle Sequencing Kit (Applied Biosystems, Warrington, UK) and products analyzed on the ABI Prism^®^ 310 Genetic Analyzer (Applied Biosystems). Data were collected with ABI Prism 310 software (Applied Biosystems), and the results analyzed with the ABI Prism DNA sequencing analysis software (Applied Biosystems). Sequence data were analyzed utilizing the Gene Runner software tool.

**The MLPA** was applied for the detection of large deletions and insertions. The probe-mixes labelled as P003 for *MLH1* and *MSH2*, P045-B1 for *BRCA2*, and P002 for *BRCA1* from MRC Holland (MRC Holland, Amsterdam, Netherlands) were used, according to manufacturer’s instructions.

### Results of genetic investigations

#### BRCA1 in BRCA2

With MLPA, DGGE and direct sequencing we did not find any changes in the sample. We detected some polymorphisms at the frequently polymorphic loci but no mutations.

However, we found a rare polymorphism in *BRCA2* gene p.V1269V which did not code a different amino acid; therefore, it did not affect protein function (Table 1). The sample was also tested for the presence of mutation 1100delC in *CHEK2* gene with MLPA kit P045-B1. In the samples mutation 1100delC was not detected.

#### P53

Tumour suppressor gene *p53* was sequenced in total. No mutations were found, but some common polymorphisms were (Table 1).

#### CDKN2A

By direct sequencing of exons of *CDKN2A* gene (p16 and p14^ARF^) we detected two polymorphisms: c.1-191A>G in 5′-UTR (untranslated region) and c.471+69C>T in 35′-UTR (Table 1).

#### MLH1 in MSH2

We detected no changes by means of the MLPA. The screening of the patients’ DNA was therefore continued through HRM and sequencing. With these two methods no mutations were discovered, but we found some polymorphisms which were frequently detected in a general population. We also detected three variants of DNA which were described as variants with unknown influence on the protein function (Table 1).

## Discussion

Secondary breast cancer is the most common SN among women who have received high dose radiotherapy.^10^ However, secondary breast cancer is a rare disease in men and very little is known about its aetiology. It has been suggested that the carcinogenic effect of ionizing radiation may be similar in the male and prepubertal female breast.^11^

Beside the radiation exposure, some of the risk factors for primary MBC^6^ were also found in our patient with secondary MBC – *i.e*. hormonal imbalances and gynecomasty.

Genetic factors associated with an increased risk of primary MBC include *BRCA2* mutations that account for 4% to 14% of all primary MBC.^6^,^12^ We found no such mutations; only a rare V1269V polymorphism that does not affect protein function.

Since p53 is often mutated in female breast cancers, it could be mutated also in MBC. Even though the mutations in tumour suppressor gene *p53* are correlated with numerous malignancies^13^,^14^, no mutations were found in our patient while screening this gene. Also in MMR genes (*MLH1* and *MSH2)* and in *CDKN2A* no deleterious mutations have been detected (Table1).

We found polymorphisms in MMR genes - *MLH1* and *MSH2*, frequently detected in a general population, but also three variants (two in *MSH2* and one in *MLH1*) which are described as variants with unknown influence on the protein function. This finding could be of interest since recent reports allude that two of these variants in *MSH2* (c.2006-6T>C and p.G322D) might influence the process of cancer development. Nucleotide variant c.2006-6T>C may be associated with an increased risk for of Non-Hodgkin’s Lymphomas.^15^ Additionally, the risk of some haematological malignancies in individuals carrying c.2006-6T>C variant is increased after the treatment with alkylating agents such as procarbazine, dacarbazine, cyclophospahmide.^16^

According to some recent reports the variant p.G322D is associated with changes in *MSH2* protein function. Individuals with this variant have slightly reduced release efficiency of mismatched targeted DNA compared to the wild type.^17^

## Conclusions

To our knowledge there are no reports of genetic screening in secondary MBC. We found three unclassified variants that could be correlated with an increased risk of secondary MBC but further studies should be performed.

## Figures and Tables

**TABLE 1:** Nucleotide variations detected in male breast cancer patient

**Gene**	BIC*	HGVS nomenclature**	**Genotype**	**Clinical significance**
*MLH1*				
		c.453+79A>G	heterozygote AG	polymorphism
		c.1668-19A>G	homozygote AA	polymorphism
		c.655A>G (p.I219V)	heterozygote AG	unclassified variant
*MSH2*		c.211+9G>C	homozygote GG	polymorphism
		c.1511-9A>T	heterozygote AT	polymorphism
		c.1661+11G>A	heterozygote GA	polymorphism
		c.2006-6T>C	heterozygote TC	unclassified variant
		c.965G>A (p.G322D)	heterozygote AG	unclassified variant
*BRCA1*	2201C>T	c.2082C>T (p.S694S)	heterozygote CT	polymorphism
	2430T>C	c.2311T>C (p.L771L)	heterozygote TC	polymorphism
	2731C>T	c.2612C>T (p.P871L)	heterozygote CT	polymorphism
	3232A>G	c.3113A>G (p.E1038G)	heterozygote AG	polymorphism
	3667A>G	c.3548A>G (p.K1183R)	heterozygote AG	polymorphism
	4427T>C	c.4308T>C (p.F1436S)	heterozygote TC	polymorphism
	4956A>G	c.4837A>G (p.S1613G)	heterozygote AG	polymorphism
*BRCA2*				
	203G>A	c.1-25G>A	homozygote GG	polymorphism
	1342C>A	c.1114C>A (p.H372N)	homozygote CA	polymorphism
	3624A>G	c.3396A>G (p.L1132L)	heterozygote AG	polymorphism
	4035T>C	c.3807T>C (p.V1269V)	heterozygote TC	polymorphism
	7470A>G	c.7242A>G (p.S2414S)	heterozygote AG	polymorphism
	IVS16-14C>T	c.7806-14C>T	heterozygote TC	polymorphism
*P53*				
		c.96 + 41_56 del CCCCAGCCCTCCAGGT	homozygote	polymorphism
		c.215 C>T (p. Pro72Arg)	heterozygote CT	polymorphism
		c.782 + 72 A>C	heterozygote AG	polymorphism
		c.782 + 92 A>G	heterozygote AC	polymorphism
*CDKN2A*				
		c.1-191A>G	homozygote GG	polymorphism
		c.471+69C>T	heterozygote CT	polymorphism

* Nucleotide variations described as in BIC (Breast Cancer Information Core) database. DNA variants are numerated according to NCBI reference sequence HSU14680 for mRNA of BRCA1, or U43746 for mRNA of BRCA2. First nucleotide of mRNA is numerated as 1.

** Description of nucleotide variations is in accordance with HGVS (Human Genome Variation Society) nomenclature. DNA variants are numerated according to NCBI reference sequence NM_000249 for *MLH1*, NM_000251 for *MSH2*, NM_007294.2 for *BRCA1*, NM_000059.3 for *BRCA2*, NM_000546 for *p53* and NM_000077.3 for *CDKN2A*. First nucleotide of start codon ATG is numerated as 1.

## References

[1] Jazbec J, Todorovski L, Jereb B (2007). Classification tree analysis of second neoplasms in survivors of childhood cancer. BMC Cancer.

[2] Metayer C, Lynch CF, Clarke EA, Glimelius B, Storm H, Pukkala E (2000). Second cancers among long-term survivors of Hodgkin’s disease diagnosed in childhood and adolescence. J Clin Oncol.

[3] Boussen H, Kochbati L, Besbes M, Dhiab T, Makhlouf R, Jerbi G (2000). [Male secondary breast cancer after treatment for Hodgkin’s disease. Case report and review of the literature]. [French]. Cancer Radiother.

[4] Ozet A, Yavuz AA, Kömürcü S, Oztürk B, Safali M, Arpaci F (2000). Bilateral male breast cancer and prostate cancer: a case report. Jpn J Clin Oncol.

[5] Weiss JR, Moysich KB, Swede H (2005). Epidemiology of male breast cancer. Cancer Epidemiol Biomarkers Prev.

[6] Giordano SH, Buzdar AU, Hortobagyi GN (2002). Breast cancer in men. Ann Intern Med.

[7] Leinung S, Horn LC, Backe J (2007). [Male breast cancer: history, epidemiology, genetic and histopathology]. [German]. Zentralbl Chir.

[8] Murata H, Khattar NH, Gu L, Li GM (2005). Roles of mismatch repair proteins hMSH2 and hMLH1 in the development of sporadic breast cancer. Cancer Lett.

[9] Latz D, Alfrink M, Nassar N, Beyerle C (2004). Breast cancer in a male patient after treatment of acute lymphoblastic leukemia including total body irradiation and bone marrow transplantation. Onkologie.

[10] Zebic-Sinkovec M, Kadivec M, Podobnik G, Skof E, Snoj M (2010). Mammographycally occult high grade ductal carcinoma in situ (DCIS) as second primary breast cancer, detected with MRI: a case report. Radiol Oncol.

[11] Thomas DB, Rosenblatt K, Jimenez LM, McTiernan A, Stalsberg H, Stemhagen A (1994). Ionizing radiation and breast cancer in men (United States). Cancer Causes and Control.

[12] Eeles RA (2000). Future possibilities in the prevention of breast cancer: intervention strategies in BRCA1 and BRCA2 mutation carriers. Breast Cancer Res.

[13] Malkin D (2001). The role of p53 in human cancer. J Neurooncol.

[14] Zager V, Cemazar M, Hreljac I, Lah TT, Sersa G, Filipic M (2010). Development of human cell biosensor system for genotoxicity detection based on DNA damage-induced gene expression. Radiol Oncol.

[15] Cesar Paz-y-Mino, Perez CJ, Fiallo BF, Leone PE (2002). A polymorphism in the *hMSH2* gene (gIVS12-6T>C) associated with non-Hodgkin lymphomas. Cancer Genet Cytogenet.

[16] Worrillow LJ, Travis LB, Smith AG, Rollinson S, Smith AJ, Wild CP (2003). An intron splice acceptor polymorphism in *hMSH2* and risk of leukemia after treatment with chemotherapeutic alkylating agents. Clin Cancer Res.

[17] Ollila S, Delmadi Bebek D, Jiricny J, Nystrom M (2008). Mechanisms of pathogenicity in human *MSH2* missense mutants. Human Mutation.

